# Synthesis of Cobalt Oxides Thin Films Fractal Structures by Laser Chemical Vapor Deposition

**DOI:** 10.1155/2014/685270

**Published:** 2014-01-05

**Authors:** P. Haniam, C. Kunsombat, S. Chiangga, A. Songsasen

**Affiliations:** ^1^Department of Physics, Faculty of Science, Kasetsart University, Bangkok 10900, Thailand; ^2^Department of Chemistry, Faculty of Science, Kasetsart University, Bangkok 10900, Thailand

## Abstract

Thin films of cobalt oxides (CoO and Co_3_O_4_) fractal structures have been synthesized by using laser chemical vapor deposition at room temperature and atmospheric pressure. Various factors which affect the density and crystallization of cobalt oxides fractal shapes have been examined. We show that the fractal structures can be described by diffusion-limited aggregation model and discuss a new possibility to control the fractal structures.

## 1. Introduction

Cobalt oxides have attracted a considerable amount of attention in the recent years for extensive applications including electronics and sensing [1, 2], catalysts [3], and biomedical devices [4, 5] due to their superior properties, such as high saturation magnetization [6, 7], stable catalytic activity [8], and excellent electrochemical performance [9, 10]. Cobalt and cobalt oxides films have been synthesized by a variety of different methods including spray pyrolysis [11, 12], electron-beam evaporation [13], sol-gel technique [14, 15], and more recently chemical vapor deposition (CVD) [16]. In fact, CVD is the preferred method for direct deposition of thin films of metals on specific structures and substrates. Laser chemical vapor deposition (LCVD) is a variation of CVD that uses a beam of laser to heat the surface of a substrate to a suitable temperature for localized synthesis. The advantage of LCVD over conventional CVD is the ability to instantly modify and control the growth rate.

Efforts have been focused recently on understanding the growth mechanisms of CoO and Co_3_O_4_ nanoparticles (NPs) in order to control and modify the structure and properties for both scientific and application purposes [17–19]. Novel properties have been promised when such NPs consist of differing nanostructures [20]. In fact, fractal structures offer a number of possibilities for the design and fabrication of novel materials and devices with unique properties. Recently, the synthesis of cobalt oxide flower-like NPs using hydrothermal process for biosensors applications has been reported [21]. In addition, the physical properties of cobalt oxide fractal structures have been investigated more recently [22].

In this paper, a procedure for producing of self-aligned CoO and Co_3_O_4_ fractal structures using LCVD method and multilayered metal thin films is reported. We will show that the size and morphology of the structures can be controlled by the laser irradiation time and the gas flow ratio, which can be optimized to exhibit fractal properties. We subsequently calculate the fractal dimensions of the growth patterns using the box-counting method [23] and compare the result with diffusion-limited aggregation (DLA) model [24]. The present results offer new prospects to control self-aligned microstructures and optimize material behavior in a wide range of applications.

## 2. Experiment

The multilayer thin films were deposited onto silicon substrates (5.0 × 5.0 × 0.5 mm^3^). Chromium (Cr) was deposited first, followed by aluminum (Al), and finally cobalt (Co). The Cr and Al were deposited using direct-current magnetron sputtering, whereas the Co was deposited by radio-frequency sputtering. In both cases, the argon flow rate was set at 125 standard cubic centimeters per minute (sccm), and the base pressure was 3.0 × 10^3^ mbar. The thickness of each layer was examined by the field emission scanning electron microscope (FESEM, JEOL JSM-7001F) which was 10 nm Co, 60 nm Al, and 170 nm Cr. The LCVD reactor consisted of a glass tube of 7.0 cm diameter and 20 cm long and a gas inlet tube of 6.35 mm diameter used to inject the gas across the deposition zone. The substrate with the Co/Al/Cr thin films was located at 5.0 mm from the inlet tube and was irradiated locally on the front surface using an unfocused beam of a 660 nm 100 mW continuous laser diode for 3.0, 5.0, and 15.0 min. The glass tube was evacuated down to a base pressure of 10^−2^ Torr by a rotary pump and brought back up to atmospheric pressure under a mixture of acetylene (C_2_H_2_) and hydrogen (H_2_). The H_2_ was maintained constant at 200 sccm, whereas the flow rate of C_2_H_2_ was 50 and 100 sccm. After laser irradiation, the samples were analyzed by the FESEM and the X-ray diffraction (XRD, Cu K*α* radiation, *λ* = 1.5640 Å, Bruker D-8).

The morphology of the cobalt oxides thin film revealed by FESEM data suggests the fractal structures. In order to confirm and understand the fractal mechanism of the as-grown films, the standard diffusion-limited-aggregation (DLA) at multiple growth sites and box-counting methods have been employed. In DLA analysis, following algorithm has been employed. Initially 15 seed particles are randomly distributed on a square lattice of 500 × 500 with periodic boundary condition. A particle is then released from a random position on the lattices and performs a random walk until it comes into contact with the growing clusters or seeds. A new particle is then released after the former particle becomes a part of the growing clusters. This process is repeated until the desire random fractal is achieved. The fractal dimension (*d*_*f*_) can be determined by the equation *R* ~ (*N*)^1/*d*_*f*_^, where *N* is the number of particles in the aggregate and *R* is the radius of gyration [24]. In the box-counting method, the leaf-like shape is covered with square boxes and counts the number of boxes necessary to cover the entire structure. The fractal dimension is then evaluated from the slope of graph of the logarithmic plot of the number of boxes versus the inverse of box size as the box size is reduced.

## 3. Results and Discussion 

**Figure 1:**
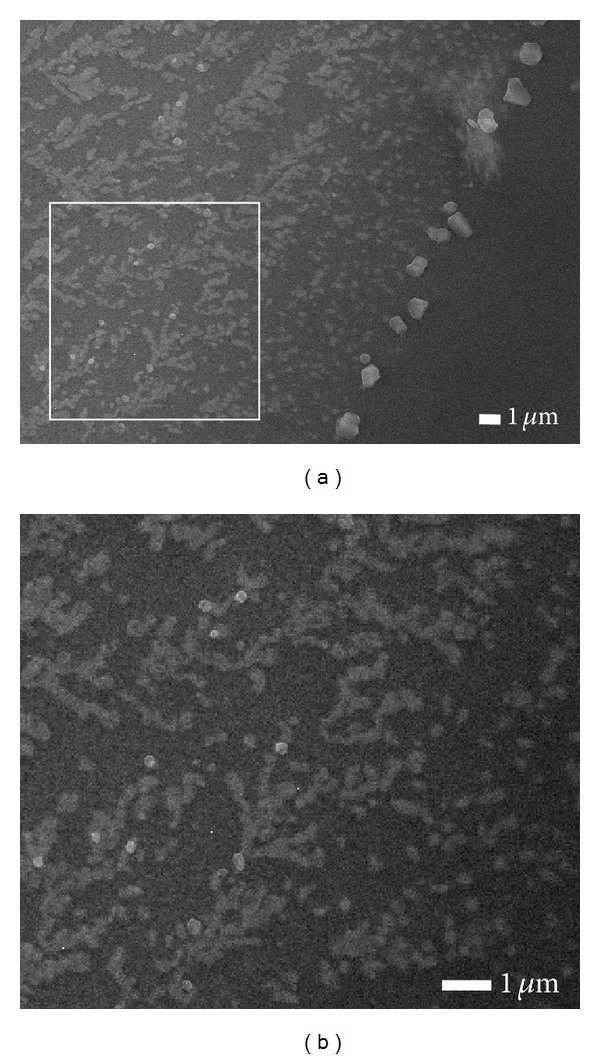
(a) Low magnification FESEM image of the surface morphology from different regions of cobalt oxide film for laser-irradiated period of 3 min with a C_2_H_2_: H_2_ gas flow ratio of 50 : 200 sccm and (b) high magnification of region in (a).

Figure 1 illustrates the effect of the thermal gradients on the resulting morphology of the cobalt oxides film. The film consists of islands, nanoparticles, and smooth region. An early state of the fractal formation by cobalt oxides islands is observed at the edge of irradiated area as shown in Figure 1(a). The NPs at a rim of irradiated region are larger than those at the inner area and on the islands. We anticipate that those results are due to the thermal gradients.

**Figure 2:**
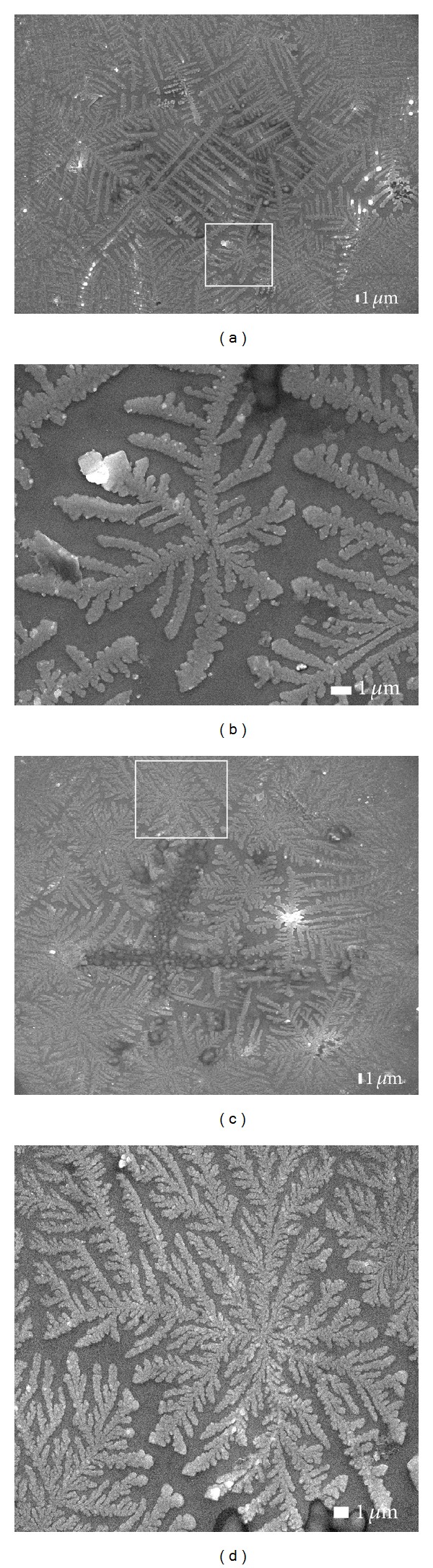
Low and high magnification of FESEM images of structures on the film surface with the same C_2_H_2_ : H_2_ gas flow rate of 50 : 200 sccm for different laser-irradiated times of (a, b) 3 min and (c, d) 5 min.

The SEM images in Figure 2 show the surface morphologies for two different irradiated periods with the same gas flow ratio. The evolution of the leaf-like structures manifests itself as an increase of irradiation time. An increase in laser exposure time from 3 min to 5 min, the leaflike structures are branched in various directions. The analysis of the leaf-like structures in Figures 2(b) and 2(d) shows that the length of main stems ranges between 2.3–9.3 *μ*m and 4.3–10.3 *μ*m, respectively.

**Figure 3:**
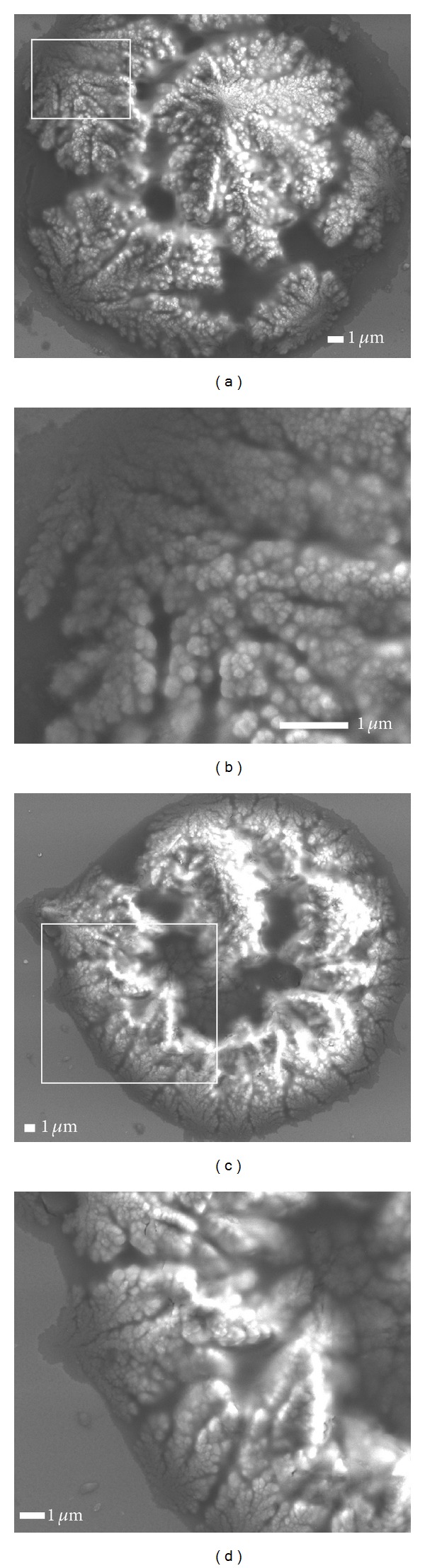
Sequence of low and high magnification of FESEM images of structures on the film surface with the same C_2_H_2_ : H_2_ flow rate of 100 : 200 sccm for different illumination of (a, b) 5 min and (c, d) 15 min.

The morphological changes of the as-grown films depend dramatically on the C_2_H_2_ : H_2_ gas flow ratio as illustrate in Figures 2(c), and 3(a). In Figure 2(c) irradiated for 5 min and at the C_2_H_2_ : H_2_ flow ratio of 50 : 200 sccm, the film surface exhibits a two-dimensional structure. An increase in the flow ratio from 50 : 200 to 100 : 200 sccm for the same exposure time of 5 min results in a three-dimensional structure of the leaf-like (Figure 3(a)).  Figure 3(b) depicts the aggregation of cobalt oxide NPs for 15 min laser exposure time, while the C_2_H_2_ : H_2_ flow ratio was kept constant at 100 : 200 sccm.

**Figure 4:**
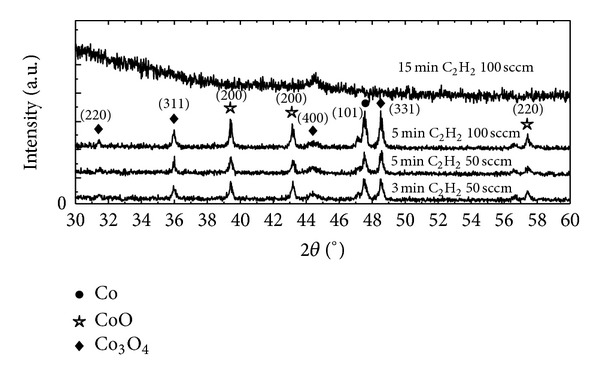
Overlay XRD patterns of leaf-like structures for three different illumination at two different gas flow rates.

Figure 4 shows X-ray diffraction patterns of leaf-like structures for three different illumination (3, 5, and 15 min) at two different C_2_H_2_ : H_2_ flow ratios of 50 : 200 and 100 : 200 sccm. The XRD analysis reveals the presence of CoO and Co_3_O_4_ phases, as well as Co crystal lattice. As shown in Figure 4, similar patterns are observed for irradiation time less than 15 min, quite independent of the gas flow ratios. The observed peak at 31.4, 36.2, 44.9, and 48.5 corresponds to the scattering from 220, 311, 400, and 331 planes of the spinel Co_3_O_4_ crystal lattice (JCPDS no. 43-1003). The CoO peaks are observed at scattering angles 39.5 and 57.4 matched to diffraction from 200 and 220 lattice planes (JCPDS no. 43-1004). The diffraction peaks at scattering angles of 47.5 are assigned to scattering from the 101 plane of the hexagonal Co crystal lattice (JCPDS no. 05-0727). This result provides that NPs having the same crystal structure as bulk Co have been formed which is in agreement with other reports [25, 26]. It is known that the cobalt oxides can be transformed into pure metallic cobalt by hydrogen reduction as described by the following equations:

(1) Co3O4+4H2⟶3Co+4H2O  (g)CoO+H2⟶Co+H2O  (g)

The XRD result indicates that prolonging the laser irradiation to 15 min at gas flow ratio of 50 : 100 sccm yields a broadened amorphous film, which is consistent with those of other studies [27, 28]. We note that the Co_3_O_4_ peak (the 400 lattice plane) with less intensity is still clearly seen for illumination of 15 min; thus, this peak may be used as a tool for detecting the final stage of these surface morphologies. The crystallite sizes of cobalt oxides can be calculated using the Scherrer equation: *d* = 0.9*λ*/*β*cos⁡*θ*, where *d* is the crystallite size (nm), *λ* is the X-ray wavelength, and *β* is the full width at half maximum of the diffraction peak at the diffraction angle 2*θ*. The calculated crystallite size of Co (101), CoO (200), and Co_3_O_4_ (331) for 3 min irradiation period at the C_2_H_2_ : H_2_ flow ratio of 50 : 200 sccm is 36, 41, and 40 nm, respectively.

**Figure 5:**
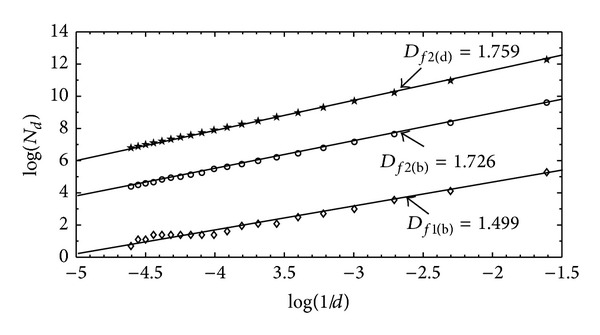
The box-counting analysis (*D*_*f*_) for a single leaf-like shape in Figures 1(b), 2(b) and 2(d) are 1.499, 1.726 and 1.759, respectively.

**Figure 6:**
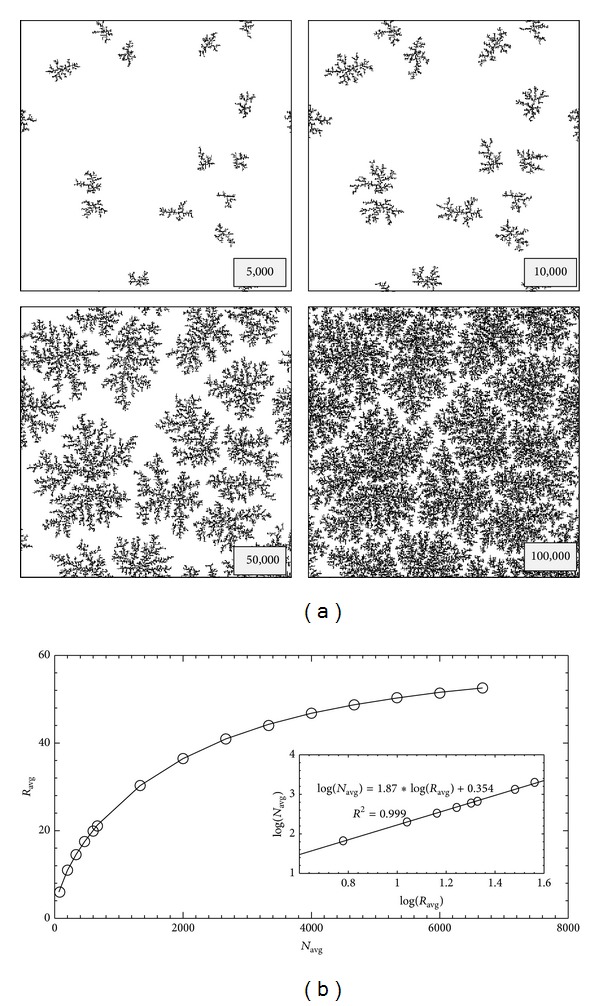
(a) Selected DLA simulation patterns for 500 × 500 lattices, 15 seed particles with four different numbers of particles, and (b) the average radius of gyration (*R*_avg_) as a function of average number of particles (*N*_avg_). The inset is a plot of the average radius of gyration and the average number of a single leaf-like shape.

The fractal nature of the surface structures was determined by using box-counting method. The analysis of the digitized images of the leaf-like shape is presented in Figure 5. The fractal dimensions (*d*_*f*_) of a single island in Figures 1(b), 2(b), and 2(d) are 1.499, 1.726, and 1.759, respectively. The obtained *d*_*f*_ values indicate that the leaf-like structures are formed by the influence of thermal force field independent of the growth conditions [23]. In order to understand quantitatively the aggregation mechanism of the NPs, the DLA simulation was performed for 500 × 500 lattices with periodic boundary condition and 15 seed particles.  Figure 6(a) shows the selected patterns for four different numbers of particles. As seen, the leaf-like shape grows initially from the islands and subsequently widens the island branches. The increase of the number of particles leads to dense islands.  Figure 6(b) presents the relationship between the average size of the islands (the average radius of gyration: *R*_avg_) and the average number of particles (*N*_avg_). This graph indicates that the average fractal dimension of the clusters increases at high density and the growth of clusters slows down as the number of particles increases. The inset is the plot of log(*R*_avg_) versus log(*N*_avg_) for a single leaf-like shape with the average number of 2,000 particles that shows the average value of *d*_*f*_ = 1.87.

## 4. Conclusion

Laser chemical vapor deposition provides an easy pathway for the synthesis of self-aligned CoO and Co_3_O_4_ fractal structures. The synthesis fractal structures consist of the cubic CoO and Co_3_O_4_ phases as well as hexagonal Co phase. The density and crystallization of cobalt oxides fractal shapes are controllable by variation of the gas flow ratios and the laser irradiation times. The fractal nature of the observed structures can be described by DLA model. The present work demonstrates new possibilities to control fractal structures, which could have an important effect on material behavior in a wide range of applications.
